# Mass Production of 3D Connective Graphene Networks by Fluidized Bed Chemical Vapor Deposition and Its Application in High Performance Lithium-Sulfur Battery

**DOI:** 10.3390/nano12010150

**Published:** 2021-12-31

**Authors:** Rongzheng Liu, Jian Zhao, Xu Yang, Malin Liu, Jiaxing Chang, Youlin Shao, Bing Liu

**Affiliations:** Institute of Nuclear and New Energy Technology, Tsinghua University, Beijing 100084, China; jian-zha20@mails.tsinghua.edu.cn (J.Z.); changjiaxing@tsinghua.edu.cn (J.C.); shaoyoulin@mail.tsinghua.edu.cn (Y.S.); bingliu@tsinghua.edu.cn (B.L.)

**Keywords:** graphene network, CVD, electrodes

## Abstract

Three−dimensional (3D) graphene with novel nano−architectures exhibits many excellent properties and is promising for energy storage and conversion applications. Herein, a new strategy based on the fluidized bed chemical vapor deposition (FB−CVD) process was proposed to prepare 3D graphene networks (3DGNs) with various nano−architectures. Specially designed SiC−C@graphene core/shell nanoparticles were prepared taking the advantages of the FB−CVD system, and 3DGNs with hierarchical nanostructures were obtained after removing the SiC core. The 3DGNs performed well as electrodes of lithium–sulfur batteries. The C–S cathode showed good rate performance at the current density of 0.1–2.0 C, and an initial discharge capacity of 790 mAhg^−1^ cathode was achieved at a current density of 0.2 C. The Li−S batteries showed stabilized coulombic efficiency as high as 94% and excellent cyclic performance with an ultra low cyclic fading rate of 0.075% for the initial 280 cycles at a current density of 1.0 C. The improved electrochemical performance was ascribed to the enhanced conductivity by the connective graphene networks and the weakened shuttle effect by the special outer graphene layers. Mass production of the products was realized by the continuous FB−CVD process, which opens up new perspectives for large scale application of 3D graphene materials.

## 1. Introduction

Since its discovery in 2004, graphene has drawn increasing attention and has been intensively investigated due to its unique and excellent properties, such as huge specific surface area, high electrical conductivity and electron mobility, robust mechanical performance, good flexibility, high thermal conductivity, and efficient light absorption [1,2,3]. Over the past decades, graphene has been widely explored for applications in batteries, electrochemical capacitors, catalysis, hydrogen storage, environmental remediation and sensors [4,5,6,7]. However, due to the strong van der Waals interactions and high inter−sheet junction contact resistance, isolated graphene sheets usually undergo irreversible agglomeration. To avoid the restacking of graphene sheets, the two−dimensional (2D) graphene is generally assembled into three−dimensional (3D) architectures with ordered structures. Three−dimensional (3D) graphene enormously maintains properties of 2D graphene in bulk and enhances graphene utilization for practical applications. In recent years, studies on graphene have shown explosive growth, especially 3D graphene in the field of electrochemical energy storage [8,9,10].

Lithium–sulfur batteries are nowadays undergoing a tremendous number of investigations because of the highest theoretical capacity of elemental sulfur among cathode materials for secondary lithium batteries [11]. However, lithium–sulfur batteries still suffer from poor cycling life and rate performance due to the intrinsic insulate sulfur/lithium sulfides and dissolution of intermediate polysulfide species for irreversible loss [12]. In addition, robust mechanical properties are required to overcome the volume expansion of sulfur during discharge [13]. In order to fabricate cathodes that are capable of delivering electrons efficiently to sulfur, trapping the soluble polysulfides and overcoming the volume change of cathodes, considerable efforts have been devoted to designing cathode materials with mesoporous and robust architectures, including porous hollow carbon, hollow carbon nanofiber, aligned carbon nanotubes and 3D graphene [14,15,16,17,18]. Among these materials, 3D graphene networks (3DGNs) show great potential as cathodes for lithium–sulfur batteries thanks to the advantages of exceptional electronic conductivity, high mechanical strength and the ability to prevent polysulfides from dissolving [19].

Until now, several methods have been developed for fabricating 3DGNs, which could be divided into two categories: assembly of graphene sheets and direct synthesis from carbon sources [20]. Assembly of graphene sheets generally gets carried out in the liquid phase. Graphene is used in the form of graphene oxide (GO) because of the good solubility conferred by the presence of hydrophilic groups. Assembly of graphene sheets achieves by dispersing GO in a solution and then reducing GO with a variety of methods, such as chemical reduction, electrochemical reduction, and hydrothermal reduction [21,22,23,24,25]. The 3DGNs fabricated by these assembly strategies usually consist of GO or reduced GO (r−GO) and present in the form of centimeters sized bulk with large pore sizes of several micrometers. Direct synthesis of 3DGNs generally achieves by chemical vapor deposition (CVD), and 3D metal substrates are usually pre−fabricated as the catalysts and templates [26,27]. These metal−catalyzed CVD methods pay more attention to the construction of nano−architectures of 3DGNs but producing 3DGNs with nanometer sized pores is difficult. Recently, Al_2_O_3_ nanoparticles (NPs) have been used as templates for the catalyst−free CVD of graphene meso−sponge with monolayer graphene [28]. 3DGNs prepared by this means possess ultra−high specific surface area and porosity, but the robustness still could not meet the need of the electrodes for high performance lithium–sulfur batteries. A one−step growth method is proposed to prepare multilayer−graphene hollow nanospheres by preparing SiC@C core/shell nanoparticles and evaporating the SiC core under high temperature [29]. Though pre−fabricated templates is not required for this method, the lack of internal nano−architectures structure of these hollow nanospheres limits their applications. Another important aspect for the 3DGNs is their mass production. Although great efforts have been devoted in the synthesis of 3DGNs, most works gain a production scale only at the laboratory level, and few works consider the mass production of these attractive materials from the point of view of engineering.

To combine the construction of 3DGNs with hierarchical nanostructures in scientific perspective and the mass production in engineering perspective, we proposed a novel strategy by fluidized bed chemical vapor deposition (FB−CVD). SiC−C@graphene core/shell nanoparticles with novel nanostructures were first prepared via the FB−CVD process. After heat treated under high temperature and vacuum environment, high purity 3DGNs were obtained. The nano−architectures of the 3DGNs could be adjusted thanks to the different microstructures of SiC−C@graphene nanoparticles prepared at different experimental conditions. Sulfur with high loading was infiltrated into the 3DGNs via a melt−diffusion strategy to prepare the carbon/sulfur composite cathodes for lithium–sulfur batteries. The cathodes showed good discharge capacity, improved cyclic performance and excellent efficiency, demonstrating a great prospect of engineering application for the 3DGNs produced by the continuous FB−CVD process.

## 2. Methods

Preparation of the 3DGNs: The SiC−C@graphene nanoparticles were prepared by a fluidized bed chemical vapor deposition (FB−CVD) method. In a typical synthesis process, the reaction zone of the vertical fluidizing tube was heated at 1000 °C. Hexamethyldisilane (HMDS) (Innochem, Beijing, China) was used as the precursor to prepare SiC–C composite nanoparticles. HMDS was heated at 80 °C by a water bath to form vapor and then was carried into the furnace by argon. The other precursor of propylene was injected to the furnace at the same time. The products were collected by powder separator device which was connected to the gas outlet of the furnace. The obtained composite nanoparticles were then heated at 1950 °C for 3 h under a vacuum degree of 10^−3^ Pa. After heat treatment, SiC particles were evaporated and the carbon phase was remained to form 3D connective graphene net−cages. To investigate the effect of reaction atmosphere, propylene concentration and temperature on the microstructure of the products, different experimental conditions were conducted as shown in Table 1.

Fabrication of 3DGNs/Sulfur Composites: The 3D graphene net−cages powder/sulfur composite cathodes were fabricated following a typical melt−diffusion strategy. The 3D graphene net−cages powder was firstly mixed with sulfur powder (Alfa Aesar Chemical Co., Ltd, Shanghai, China) with a mass ratio of 1:4 by milling. Subsequently, the mixture was placed in a sealed flask at 155 °C for 5 h to incorporate sulfur into the 3D graphene net−cages.

Characterizations: Microstructures of the product were observed by a field emission scanning electron microscope (FESEM, ZEISS EVO18, Oberkochen, Germany). The TEM images and corresponding selected area electron diffraction (SAED) patterns were obtained on a high resolution transmission electron microscope (HRTEM, JEOL 2100, Tokyo, Japan) at an acceleration voltage of 200 kV. The crystal structure of the samples was examined by X−ray diffraction (XRD) on a diffractometer using Cu Kα radiation (Bruker D8−Advance, λ = 1.5418 Å, Berlin, Germany). Raman scattering was excited employing a He–Ne laser (λ = 516 nm), and was collected by a micro−Raman spectrometer (HR800, Horiba, Montpellier, France) in the wave number of 1000–3500 cm^−1^ at room temperature. The differential scanning calorimetry (DSC) and thermogravimetry (TG) measurements were performed by a simultaneous thermal analysis apparatus (NETZSCH−STA, 409 PC, Netzsch-Gerätebau GmbH, Selb, Germany) with a heating rate of 5 °C/min in different atmospheres.

Electrochemical Measurements: Li−S batteries were constructed to evaluate the electrochemical performance of 3D graphene net−cages as cathode scaffolds according to the procedure of the reference [30]. The cathode slurry was prepared by homogeneously mixing 70 wt % of the 3DGNs/sulfur composites, 10 wt % of the PVDF(Shenzhen Kejing Star Technology Co., Shenzhen, China) binder and 20 wt % of the CNT conductive agents in an *N*−methyl−pyrrolidone dispersant by a magnetic stirrer for 24 h. The slurry was then coated onto an aluminum foil, followed by a vacuum drying for 6 h at 60 °C to obtain an areal sulfur loading of 1.1 mg·cm^−2^. Disks of 13.0 mm were punched as the working cathodes. A quantity of 25.0 μL electrolyte containing 1.0 M lithium bis (trifluoromethanesulfonyl)imide solution in 1,3−dioxolane/1,2−dimethoxyethane (1:1 v/v) (Alfa Aesar Chemical Co., Ltd, Shanghai, China)was used to construct the Li–S cell. Lithium metal foil was used as the anode and the polypropylene membranes were used as the separators. The coin cells were tested in galvanostatic mode at various currents within a voltage range of 1.6–2.8 V using multichannel battery cycler (Neware, Hong Kong, China). The cyclic performance was tested at a current density of 1.0 C. The capacities were calculated corresponding to the total mass of the cathodes slurry.

## 3. Results and Discussion

**Figure 1:**
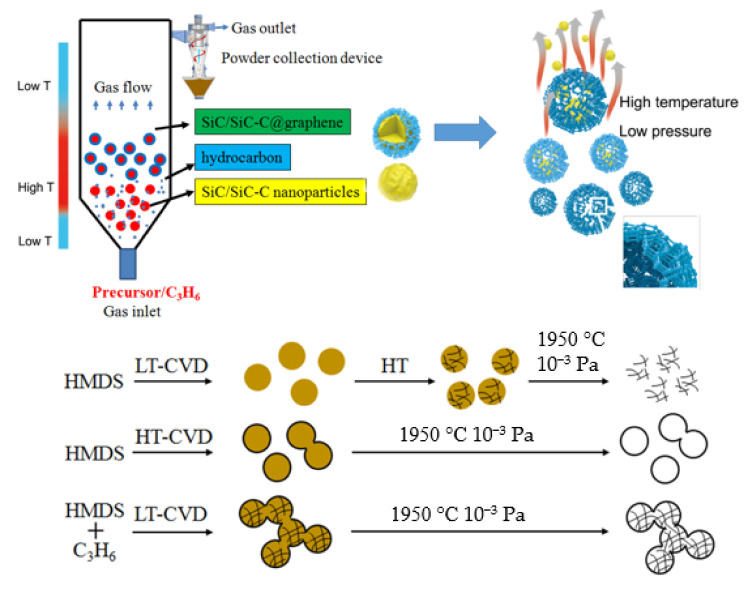
Schematic diagram of the preparation process for the 3DGNs by fluidized bed chemical vapor deposition combined with subsequent heat treatment.

The spouted fluidized bed (FB) technology and chemical vapor deposition (CVD) method are combined to prepare C–Si based nanoparticles with hierarchical structures. Compared with conventional CVD route, the so−called spouted fluidized bed chemical vapor deposition (FB−CVD) method originally used for nuclear fuel particle coating, displays many advantages in the synthesis of nanostructured particles [31,32,33,34]. In the nozzle area of the spouted fluidized bed, the gas velocity is very fast, which results in a very high nucleation rate. The quick transport process with short residence time makes the nanoparticles showing narrow size distributions and promotes the uniformity of the products. The vertical fluidized bed reactor with specially designed uneven temperature distribution also provides the possibility for in situ nano−coating by controlling the formation of the core particles and the shell layers in different deposition zones. A special SiC−C@graphene nanostructure used to prepare 3DGNs was firstly obtained by the advantage of the FB−CVD system. As illustrated in Figure 1, by using single precursor of hexamethyldisilane (HMDS) under higher Ar concentration, the excess pyrolytic hydrocarbon intermediates were favored to form free carbon. The free carbon was well mixed with the SiC phase to form amorphous SiC–C composite nanoparticles under lower synthesis temperatures, and the two phases could be distinguished after further crystallization process. By increasing the preparation temperatures, the crystallized SiC nanoparticles with smaller size was firstly formed on the bottom of the furnace and graphene layers were then coated on the surface of the SiC nanoparticles when they were transported from the low temperature zone to the high temperature zone. A combined mixing and coating process could be realized by inducing external carbon sources of propylene under proper temperatures. Thanks to the steep uneven temperature distribution of the vertical furnace, the SiC–C nanoparticles were formed when HMDS was immediately injected into the furnace at which the temperature was controlled sufficient for HMDS decomposition while inadequate for propylene pyrolysis. Carbon originated from propylene began to form when the reactants transported to the high temperature zone and was preferentially coated on the surface of the SiC–C particles by the heterogeneous nucleation mechanism. Connective 3DGNs were obtained by removing the SiC phase under the conditions of high temperature and low pressure. However, the high temperature may limit the practical applications of the nanoparticles partly.

**Figure 2:**
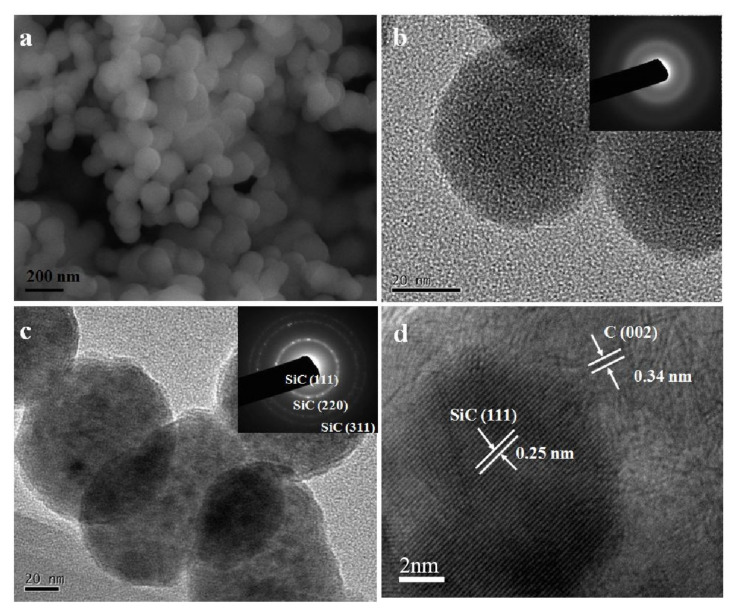
Morphologies of the SiC–C nanocomposites. (**a**) SEM and (**b**) TEM images of the SiC–C composite nanoparticles prepared at the FB−CVD temperature of 1000 °C under pure Ar atmosphere (sample S1). (**c**,**d**) HRTEM images of S1 after heat treatment at 1300 °C. The insets of (**b**,**c**) are the corresponding selected area electron diffraction (SAED) patterns of S1 before and after heat treatment.

**Figure 3:**
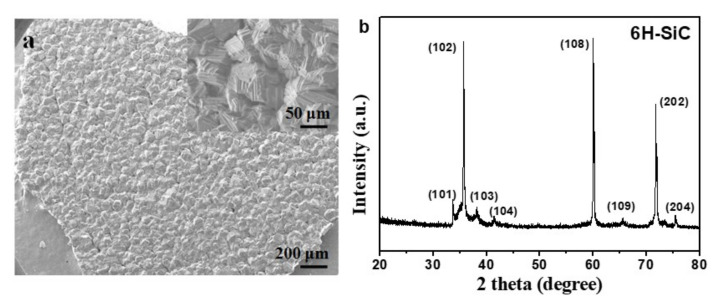
(**a**) SEM images and (**b**) XRD pattern of the deposited SiC product on the graphite crucible cover.

**Figure 4:**
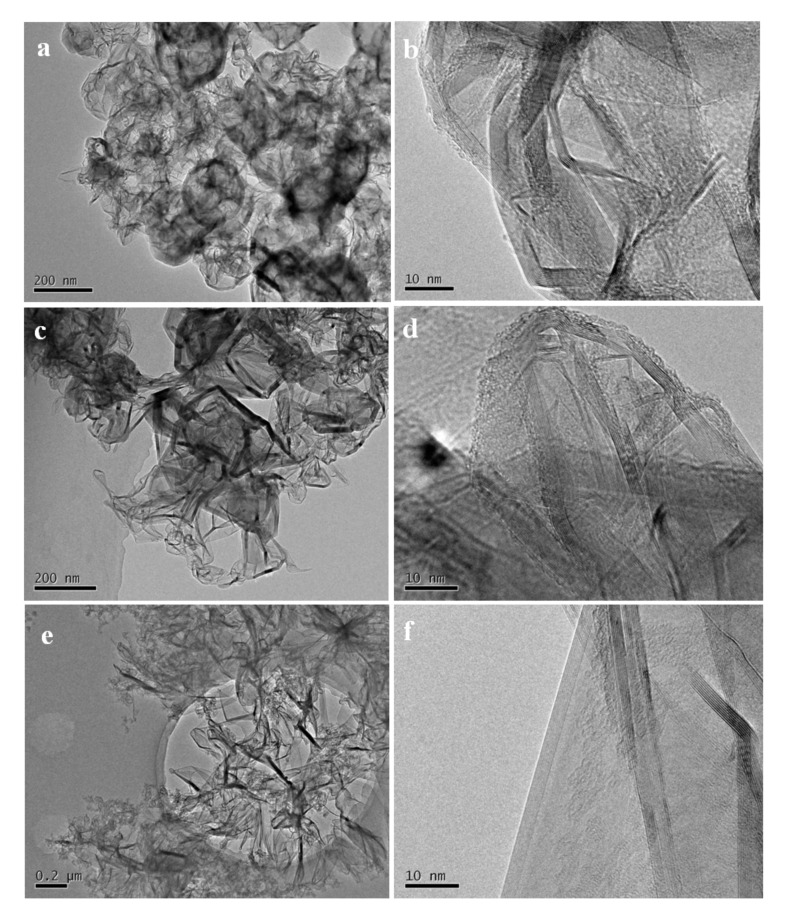
Typical TEM and HRTEM images of the graphene nanostructures obtained from SiC–C composites of: (**a**,**b**) S1, (**c**,**d**) S2 and (**e**,**f**) S3.

As shown in Figure 2a,b, the SiC–C composite nanoparticles prepared at the FB−CVD temperature of 1000 °C under pure Ar atmosphere (sample S1 from Table 1) displayed amorphous feature and the phases of SiC and C were difficult to distinguish. After heat treatment at 1300 °C, novel hierarchical structure with SiC nanoparticles embedded into the graphite matrix was obtained in the SiC–C nanospheres (Figure 2c). The SiC nanoparticles showed a size of 5~8 nm with high crystallinity as indicated by the clearly lattice fringes in HRTEM image (Figure 2d). The carbon phase as a matrix displayed a disorderly graphite structure with a typically lattice distance of 0.34 nm. The morphology and structure of the nanoparticles changed dramatically after further heat treated in a high vacuum atmosphere at temperatures higher than 1900 °C. Under such conditions, SiC nanoparticles were eliminated from the composite nanoparticles and only carbon was left. Figure 3 shows the SEM images and corresponding XRD pattern of the deposited product on the inner surface of the graphite crucible cover and well crystallized 6H−SiC (JCPDS: 75−1541) with very large grains was obtained. As the initial grain size of SiC was smaller than 10 nm and it was easily to decompose under such conditions and could be re−deposited on the cold end. Figure 4a,b show the TEM images of the high temperature treated sample from S1. The nanoparticles tended to hollow with some remained spherical cage−like 3D framework. To investigate the effect of carbon content on the final microstructure of the 3D graphene, synthesis conditions were changed with different Ar/H_2_ ratios. When 30% H_2_ was introduced into the furnace, which meant a decrease of the carbon content, the framework of the 3D graphene tended to open and lose the spherical shape, presenting a flower−like structure (Figure 4c,d). Three−dimensional (3D) graphene structure was totally transferred to 2D layered structure by further decreasing the carbon contend with increasing the H_2_ concentration to 50% for preparing the SiC–C composite nanoparticles (Figure 4e,f).

**Figure 5:**
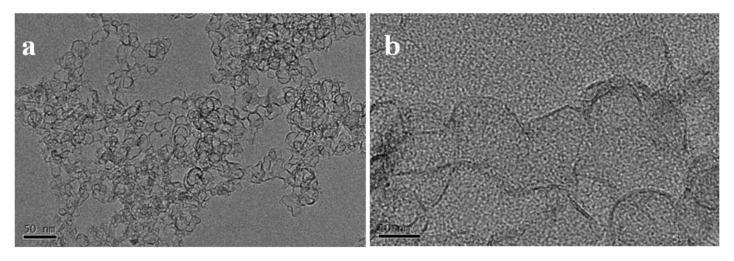
TEM images of the connective 3D graphene hollow cages obtained from the sample prepared at the FB−CVD temperature of 1300 °C under pure Ar atmosphere (sample S4). (**a**) Low magnification, (**b**) High magnification.

The results above demonstrated that the 3D framework of the graphene would lose by decreasing the content of carbon. From the experimental results, there were two ways to increase the carbon content: increasing the Ar concentration or increasing the preparation temperature. Figure 5 shows the 3D graphene hollow cages obtained for the sample prepared at the FB−CVD temperature of 1300 °C under pure Ar conditions (sample S4 from Table 1). The cages showed a totally hollow structure with very thin coatings of 2~4 graphene layers. These cages also showed a connective feature with a long through channel between particles. The carbon content of this typed SiC–C composite particles was about 6% as confirmed by TG measurement. Further increasing the carbon concentration was difficult by only using single HMDS precursor.

**Figure 6:**
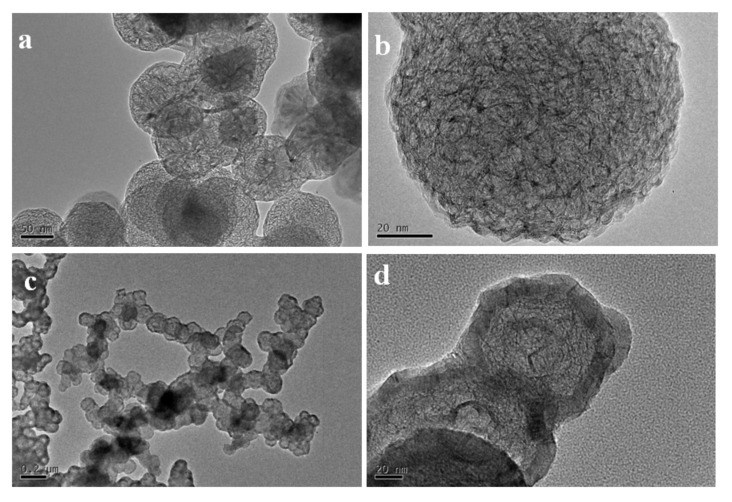
Typical TEM images of the 3DGNs obtained from SiC−C@graphene nanostructures of: (**a**,**b**) S5 and (**c**,**d**) S7.

To prepare 3DGNs showing both hierarchical internal structure and connective framework, we introduced propylene into the deposition system. From previous mechanism discussion, carbon from pyrolysis of propylene was mainly coated on the surface of the SiC–C nanoparticles as an external connector. Figure 6 shows the 3D graphene net−cages obtained with different flow ratios of the argon carrier gas and propylene. When the gas flow ratio of the carrier gas and propylene was 1:1, the 3D graphene showed an interlaced structure by graphene nanobelts (sample S5). However, as the concentration of the propylene was not so high, distribution of the particles mainly showed a dispersed feature and only a few particles connected together (Figure 6a,b). On the other hand, when higher propylene was used (carrier argon: propylene = 1:3, v/v), most particles were connected together to form a framework in a large scale. However, for such type 3DGNs, the outer graphene layers were much thick, which might weaken the effectiveness of whole particles by blocking the internal carbon from contacting with the external environments (Figure 6c,d).

**Figure 7:**
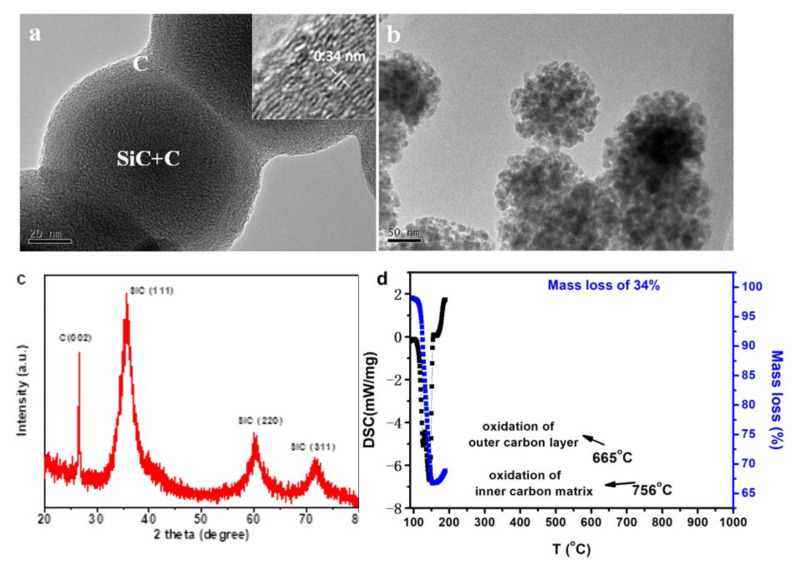
(**a**) TEM image of the SiC−C@graphene nanostructure (sample S6) and the inset shows the HRTEM image with a lattice distance of 0.34 nm for the outer carbon layer. (**b**) TEM image of the flower−like SiC nanoparticle assemblies after oxidation of the carbon phase. (**c**) XRD pattern of the SiC−C@graphene nanostructure after heat treatment at 1300 °C. (**d**) TG−DSC curves for SiC−C@graphene nanostructure measured in air atmosphere.

A balance was achieved by controlling gas flow ratio of the argon carrier gas and propylene to 1:2. Figure 7 shows some characterization results of the products obtained under such experimental condition. From TEM images of the as−obtained nanoparticles, evident carbon layer was observed on the surface of the SiC–C particles to form a connective SiC−C@graphene core−shell network (Figure 7a). From XRD pattern, very strong carbon peak was detected with the cubic SiC phase when the sample was heat treated at 1300 °C in argon atmosphere, demonstrating the existence of higher content of carbon (Figure 7c). All the SiC peaks were broadened indicating very small crystal size. From the results of TG−DSC measurements in air atmosphere (Figure 7d), the composite nanoparticles showed a total carbon weight loss of 34% before 800 °C. In the DCS curve, two exothermic peaks were observed at 665 and 756 °C, which were corresponded to the oxidation of the outer carbon layer and the inner carbon matrix, respectively. The SiC nanoparticles were revealed by oxidation of the carbon phase and a flower like assembled structure was obtained when both the external and internal carbon were oxidized (Figure 7b).

**Figure 8:**
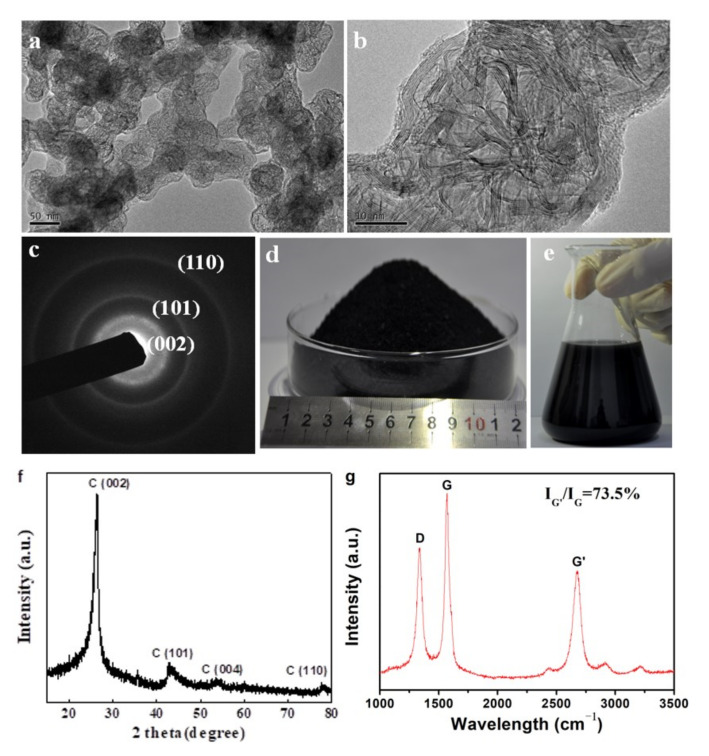
Characterizations of the 3DGNs obtained from SiC−C@graphene nanostructures of sample S6: (**a**,**b**) Typical TEM images, (**c**) corresponding SAED patterns, (**f**) XRD pattern and (**g**) Raman spectrum. (**d**) Photos of the mass−produced powder. (**e**) Photos of the products dispersed in ethanol after long time placement for one month.

Morphologies of the 3DGNs obtained from sample S6 were shown in Figure 8a. The products showed a hierarchical structure. Macroscopically, the net−cages with an average diameter of 50 nm were connected to each other to form a 3D framework. For each single net−cage, it was composed by interlaced graphene nanobelts with an evident graphene shell (Figure 8b). SAED pattern showed three polycrystalline rings corresponding to the (002), (101) and (110) planes of the graphite structure (Figure 8c). XRD pattern of the product showed typical diffraction peaks of 6H graphite structure (JCPDS: 75−0444) (Figure 8f). From the spectrum of Raman scattering, the D band, and G band located at 1330 and 1580 cm^−1^ was identified, respectively, and a strong peak at 2600 cm^−1^ appeared, which is the characteristic G’ band of graphene (Figure 8g) [35]. The D band with relatively high intensity implied that there were abundant defects in the graphene. The intensity of G’/G was as high as 73.5% indicating that the 3DGNs were composed by few−layer graphene which was consistent with the TEM observations [36,37]. By using the FB−CVD method, mass production was realized with a yield of 150 g/h (Figure 8d). As the products could be continuously collected by a powder collection equipment connected to the outlet of the furnace, the production in kilogram level per day was achieved by using medium sized fluidized reactor. The obtained 3DGNs could also be well dispersed in ethanol without evident agglomeration after a long time placement for one month (Figure 8e), which means it is very convenient to use in the future.

**Figure 9:**
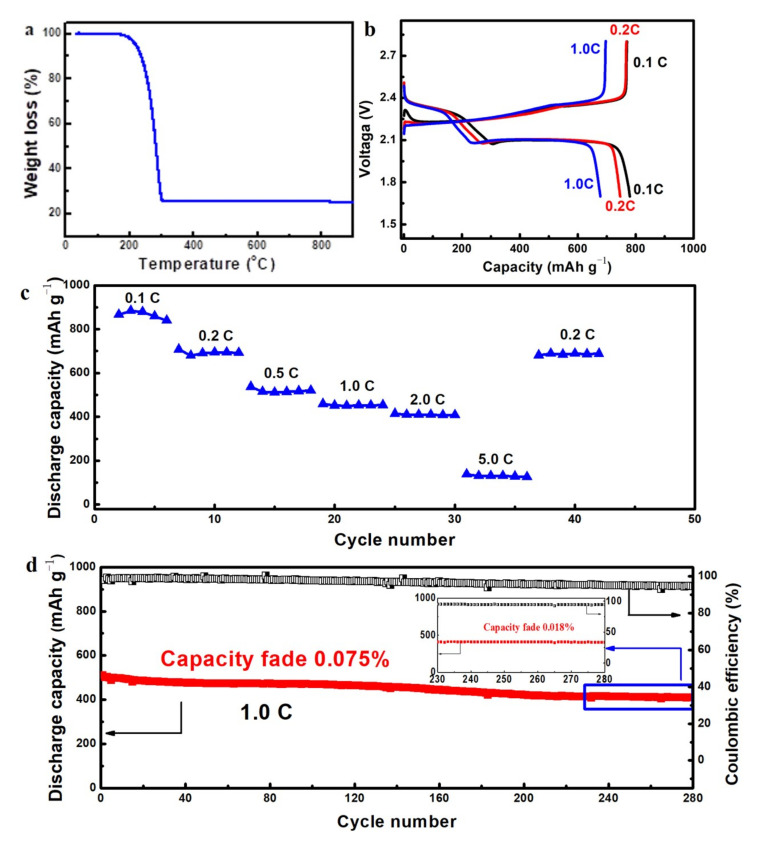
Electrochemical performance of 3DGNs/S cathodes. (**a**) TG curve of the C–S composite measured in an inert atmosphere. (**b**) Charge/discharge curves and (**c**) rate performance curves for the samples at different current densities. (**d**) The cyclic performance and coulombic efficiency of the cathode at a current density of 1.0 C.

These 3DGNs with unique microstructures will find diverse applications in the area such as lithium ion battery, catalytic carrier and supercapacitors by integrating with other functional materials. Herein we present one example for its application as the cathode scaffolds for Li–S batteries. The sulfur was infiltrated into the graphene net−cages via a melt−diffusion strategy with a C/S mass ratio of 1:4. From the TG result shown in Figure 9a, the sulfur loading in the C–S composites was 76 wt % after infiltration, which indicated that almost all the sulfur mixed was absorbed by the hollow structured 3DGNs. The high sulfur loading capacity may result from the criss−crossed carbon nanobelts filled in the net−cages which enhanced the affinity between sulfur and carbon. The Li–S battery performance was measured in standard 2025 coin cells. The capacities were calculated corresponding to the total mass of the cathodes slurry described in the experimental section. From the charge/discharge voltage curves shown in Figure 9b, an initial discharge capacity of 790 mAh/g was achieved at a current density of 0.2 C. Two typical voltage plateaus at 2.3 and 2.1 V were observed in the charge/discharge voltage curves, corresponding to a rapid reduction process of sulfur into soluble high−order lithium polysulfides and a more sluggish reaction of further lithiation into solid lithium polysulfides, respectively [38]. The discharge capacities of the C–S cathode gradually decrease with increasing the current rates as shown in the rate performance curve (Figure 9c). The C–S cathodes showed good rate performance at the current density of 0.1–2.0 C. When the current density was increased to 5.0 C, a significant discharge capacity decrease was observed, which might be attributed to the low conversion rate from polysulfide to lithium sulfide under such high current density. The cyclic stability was investigated at a current density of 1.0 C as shown in Figure 9d and an ultra−low cyclic fading rate of 0.075% for the initial 280 cycles was achieved. The cathode seemed more stable in the last 50 cycles and a cyclic fading rate of 0.018% was obtained. The coulombic efficiency of the cathode was also stabilized as high as 94% during the whole cycles.

According to the electrochemical results above, we believe that the good discharge capacity, excellent cyclic performance and high efficiency are ascribed to the high affinity of C and S which originates from the hierarchical nanostructure of 3DGNs. The connective graphene networks improve the conductivity of the C–S cathode which is beneficial to increase the discharge capacity. The interlaced internal graphene nanobelts in the hollow spheres are robust enough to resist volume change and can also provide ideal interior environment for the electrochemical conversions. The special outer graphene layers limit the shuttle effect by hindering the migration of polysulfides to electrolyte which is considered to be the main factor for the deterioration of cyclic performance and efficiency of lithium–sulfur batteries.

## 4. Conclusions

Three−dimensional (3D) graphene networks with novel nano−architectures were prepared by combining the FB−CVD process and subsequent heat treatment. Taking the advantages of the uneven temperature distribution of the fluidized bed and the rapid transport mechanism, core/shell structured SiC−graphene nanocomposites were prepared. The 3DGNs with different nano−architectures could be fabricated by adjusting the reaction atmosphere, temperature and precursors of FB−CVD. Novel 3D graphene net−cage nanostructures with interlaced graphene nanobelts and connected graphene shells were obtained from SiC−C@graphene nanocomposites. The 3DGNs performed well as electrodes of lithium–sulfur batteries with high sulfur loading, high discharge capacity, excellent cyclic performance and high efficiency. The hierarchical internal and external microstructures of the 3DGNs were responsible for the high electrochemistry performance by improving the conductivity, resisting the volume change and hindering the shuttle effect. This work provides a novel and effective synthesis strategy for the preparation 3DGNs with nano−architectures. These unique 3DGNs will find diverse applications in areas such as lithium−ion batteries, catalytic carriers and supercapacitors by fine structure design and integration with other functional materials. The realization of mass production by the FB−CVD process opens up new perspectives for large scale engineering application of the 3D graphene materials.

## Figures and Tables

**Table 1:** Typical FB−CVD conditions to prepare the composite nanoparticles.

	S1	S2	S3	S4	S5	S6	S7
Fluidizing Ar gas flow (L/min)	3.7	2.5	1.7	3.7	3.4	3.1	2.8
Fluidizing H_2_ gas flow (L/min)	0	1.2	2.0	0	0	0	0
Carrier Ar gas flow (L/min)	0.3	0.3	0.3	0.3	0.3	0.3	0.3
Propylene gas flow (L/min)	0	0	0	0	0.3	0.6	0.9
FB−CVD temperature (°C)	1000	1000	1000	1300	1000	1000	1000

## Data Availability

Data is contained within the article.
